# TRH can stimulate the release of two POMC-derived pituitary hormones, ACTH and MSH, in medaka

**DOI:** 10.1210/endocr/bqag037

**Published:** 2026-03-30

**Authors:** Mana Yamakawa, Deodatta Shyam Gajbhiye, Matan Golan, Shinji Kanda

**Affiliations:** Atmosphere and Ocean Research Institute, The University of Tokyo, Kashiwa, Chiba 277-8564, Japan; Department of Animal Sciences, The Robert H. Smith Faculty of Agriculture, Food and Environment, The Hebrew University of Jerusalem, Rehovot 7610001, Israel; Department of Animal Sciences, The Robert H. Smith Faculty of Agriculture, Food and Environment, The Hebrew University of Jerusalem, Rehovot 7610001, Israel; Atmosphere and Ocean Research Institute, The University of Tokyo, Kashiwa, Chiba 277-8564, Japan

**Keywords:** TRH, ACTH, MSH, teleost, HPA axis, pituitary

## Abstract

Anterior pituitary hormone secretion is generally considered to be under the strong regulation of hypothalamic neuropeptides. In mammals, adrenocorticotropic hormone (ACTH), which plays a crucial role in the stress response, is secreted from corticotropes and is regulated primarily by corticotropin-releasing hormone (CRH). In teleosts, although the pharmacological effects of hypothalamic factors have been demonstrated, their relative importance in regulating ACTH release remains controversial. One reason for this is the lack of methods for evaluating ACTH release at cellular resolution. Using medaka as a model organism, we systematically examined the direct effects of hypothalamic peptides on ACTH cells by combining cell type-specific transcriptomics with Ca^2+^ imaging. We show that thyrotropin-releasing hormone (TRH) robustly elevates intracellular Ca^2+^ concentration ([Ca^2+^]ᵢ) in ACTH cells, surpassing the responses elicited by CRH or arginine vasotocin (AVT). TRH also strongly activates MSH cells, the other POMC-derived pituitary cell population, while CRH induces only a modest response. Furthermore, *in situ* hybridization chain reaction analyses revealed that TRH receptor (*trhra*) is expressed in MSH cells, supporting their direct responsiveness to TRH signaling, whereas TRH receptor expression in ACTH cells was below the detection limit, leaving open the possibility that their activation is mediated by indirect or low-abundance receptor pathways. These findings may suggest the existence of a novel TRH-driven regulatory pathway orchestrating both the stress axis and the pigmentation axis.

Nearly two decades after the theoretical framework of physiological stress responses was proposed (1), a model in which the secretion of anterior pituitary hormones is regulated by hypothalamic factors was proposed (2). Subsequent studies demonstrated that humoral signals derived from the hypothalamus stimulate anterior pituitary hormone secretion, including adrenocorticotropic hormone (ACTH) release, thereby providing experimental evidence for hypothalamic-pituitary regulation (3, 4). Furthermore, the identification of corticotropin-releasing hormone (CRH; originally termed corticotropin-releasing factor [CRF]) from sheep (5) and the demonstration that CRH induces ACTH secretion defined the Hypothalamic-Pituitary-Adrenal (HPA) axis as the physiological center of the stress response and elucidated its molecular basis (6).

In teleosts, ACTH stimulates cortisol secretion from interrenal cells, the functional homolog of the adrenal cortex (7, 8), forming a hypothalamic–pituitary–interrenal axis analogous to the mammalian HPA axis. Although CRH and arginine vasotocin (AVT, the teleost homolog of vasopressin) are reported to be possible hypothalamic regulators of ACTH release, their effects differ substantially across species and experimental contexts (9‐11). Similarly, other vertebrates also present a complex, diverse regulatory system of ACTH secretion. In amphibians, for example, AVT rather than CRH has been reported to play a dominant role in stimulating ACTH release, whereas CRH primarily promotes thyrotropin (Thyroid-Stimulating Hormone, TSH) secretion (12‐15). In reptiles, experimental evidence for direct ACTH regulators remains limited. Culture experiments using turtle pituitaries demonstrated that TRH and CRH stimulated TSH and growth hormone secretion, suggesting that hypophysiotropic regulation in reptiles may differ from that in mammals (16). In birds, ACTH secretion is controlled by CRH similarly to that in mammals, but AVT alone or in combination with CRH has been shown to exert a prominent stimulatory effect (17‐19). These findings indicate that the regulatory system of ACTH release in vertebrates is diverse. Thus, studying teleosts, which diverged early from the tetrapod lineage, offers a unique opportunity to uncover alternative molecular pathways governing ACTH secretion and to reassess the evolutionary trajectory of vertebrate stress axis regulation.

To assess hormone release, transgenic model fish expressing a Ca^2+^ indicator in specific endocrine cells provide a hypothalamus-independent *ex vivo* experimental system that allows the monitoring of a large population of cells within the whole-organ context with single-cell spatial and sub-second temporal resolutions (20‐22). Additionally, cell type-specific transcriptomics or single-cell transcriptomes (23, 24) have enabled the identification of receptors expressed in each endocrine cell type (25). By applying these approaches, we can now search for candidate hypothalamic factors, examine their effects on individual ACTH cells and MSH (melanocyte-stimulating hormones) cells, and explore their neuropeptidergic regulation. Although CRH and AVT have long been hypothesized as primary hypothalamic regulators of ACTH secretion in teleosts, our findings indicate that ACTH regulation may involve additional hypothalamic mechanisms beyond these classical factors. Indeed, previous studies have suggested the presence of supplementary neuropeptidergic inputs to ACTH cells (11). Therefore, our aim was to revisit this long-standing hypothesis by combining ACTH cell-specific transcriptomics with Ca^2+^ imaging. This approach enabled a systematic search for possible hypothalamic regulators, including previously unrecognized regulators. Ca^2+^ imaging analysis of the response of ACTH cells to synthetic peptides corresponding to the detected receptors enabled the *ex vivo* validation of candidate molecules as ACTH secretagogues. Furthermore, by extending our analysis to MSH cells, we investigated whether the release of different proopiomelanocortin (POMC)-derived hormones, ACTH and MSH, is controlled by distinct or the same sets of hypothalamic modulators.

## Methods

### Animals

Medaka (*Oryzias latipes*; RRID: NCBITaxon_8090) were maintained and used in accordance with the guiding principles for the Care and Use of Experimental Animals of the University of Tokyo (Permission number: P25-10). *pomca*:GCaMP medaka, which specifically express GCaMP6s in ACTH and MSH cells (26), were used in Ca^2+^ imaging. For histological analysis, himedaka strain or its substrain, the d-rR strain, was used. The fish were maintained under a 14-hour light/10-hour dark cycle at 27 °C and fed twice daily with live brine shrimp and flake food, except on Sundays. Adult fish (>3 months old) were used unless otherwise noted. In this study, we used both females and males, and the number of samples of each sex is indicated mainly in figure legend. Because we found no obvious differences between sexes in each experiment, we pooled data from males and females.

### Transgenic line

The *pomca*:GCaMP6s transgenic medaka line labels POMC-expressing cells in the pituitary, including corticotropes and melanotrophs. This line was originally established in the himedaka background and was maintained by breeding for 16‒20 generations. Founders were screened by DsRed fluorescence driven by a larval-stage globin enhancer, and genotypes were confirmed by pituitary-specific GCaMP fluorescence. Because melanotrophs expressing Opn5m autonomously increase their [Ca^2+^]_i_ during imaging (26), the *pomca*:GCaMP6s line was crossed with the Opn5m knockout (KO) background; all Opn5m KO lines used in this study were previously generated and characterized.

### Low-input RNA sequencing analysis

Adult *pomca:GCaMP6s* medaka were used for bulk ACTH cell RNA sequencing. Corticotrope populations in the rostral pars distalis were visually identified under a fluorescence microscope (Leica M165 FC, Leica Microsystems, Wetzlar, Germany). After pituitary dissection, the pituitaries were treated with 0.05% collagenase (FUJIFILM Wako Pure Chemical Corporation, Osaka, Japan; Cat. No. 034-22363) in artificial cerebrospinal fluid (aCSF: NaCl 134 mM, KCl 2.9 mM, HEPES 10 mM, MgCl_2_ 1.2 mM, CaCl_2_ 2.1 mM, glucose 15 mM, pH 7.35-7.45) on ice for 20 minutes, and the cells were manually dissociated via the tip of a glass capillary. Isolated GCaMP-positive cells were washed at least three times with ice-cold phosphate-buffered saline (PBS; 137 mM NaCl, 2.7 mM KCl, 10 mM Na_2_HPO_4_, and 1.8 mM KH_2_PO_4_, pH 7.4) to minimize contamination. Approximately 10-20 GCaMP-positive cells per sample were collected (n = 9, pooled samples from mixed-sex fish; each sample includes cells pooled from 10-20 individuals). RNA-seq libraries were prepared with NEBNext® Single Cell/Low Input RNA Library Prep Kit for Illumina® (New England Biolabs, Ipswich, MA; Cat. No. E6420S) according to the manufacturer's instructions. Library quality and fragment size (mean 320-520 bp) were evaluated with an Agilent 4200 TapeStation system (Agilent Technologies, Santa Clara, CA) according to the manufacturer's instructions. Libraries were sequenced by Nippon Genetics (Tokyo, Japan) on an Illumina NovaSeq 6000 (Illumina, San Diego, CA) with paired-end 150 bp reads, yielding approximately 2 GB per sample. The sequencing reads were mapped to the medaka reference genome (*Oryzias latipes*, Ensembl release 114) using STAR v2.5.4b (27) with default parameters, and the read counts were quantified using RSEM v1.3.3 (28) with default settings. Gene expression levels were normalized to transcripts per million (TPM). In the next step, to exclude samples with amplification bias or contamination from other cell types, we selected data based on the following criteria: 1. the TPM value of *pomca* is above 10 000; 2. the TPM of other pituitary hormones is below 100 000; and 3. the number of detected genes is above 1000. Resulting three samples (designated as Sample A-C) were used for further analysis. Candidate receptors previously reported to mediate ACTH stimulation (29) were selected for further analysis on the basis of evidence from the literature.

### Ca^2+^ imaging experiments

Adult *pomca:*GCaMP6s medaka (93-199 mg) and *opn5m* KO:*pomca:*GCaMP6s medaka (64-87 mg) were anesthetized with 0.02% MS-222 and decapitated. The pituitary was isolated and placed in a recording chamber with a volume of approximately 300 µL. Peptide solutions were perfused at room temperature using a peristaltic pump (Rainin Dynamax RP-1, Rainin, Columbus, OH) at a flow rate of approximately 350 µL/min. Ca^2+^ imaging was performed using an upright fluorescence microscope (Eclipse E600FN; Nikon, Tokyo, Japan). A light source, X-Cite 110LED illumination system (Excelitas Technologies, Waltham, MA) and a filter (EX; 450-490, Dichroic Mirror; 505, BA; 520 [LP]) were used for GCaMP excitation. Fluorescence images were acquired every 3 second using a scientific CMOS camera (Andor Zyla 4.2 PLUS, Oxford Instruments, Belfast, UK) with 4 × 4 binning and 40 ms exposure and an LED excitation source (X-Cite 110, Excelitas Technologies, Waltham, MA). Image acquisition was controlled with MicroManager v1.4. The data were saved in 16-bit TIFF format.

To assess concentration-dependent responses, TRH (synthetic, ≥99% HPLC; Peptide Institute, Osaka, Japan) was diluted to 1000, 100, 10, 1, and 0.1 nM in aCSF. CRH (ovine; Peptide Institute, Osaka, Japan) and AVT (gift from Prof. Umatani, Tokyo University of Agriculture and Technology) were diluted to 100 nM. Peptide application sequences were counterbalanced across preparations to minimize order effects. Each peptide was applied once per preparation, followed by a 10-15 minutes aCSF wash during continuous Ca^2+^ imaging. Although the peptide concentration of 100 nM is slightly higher than the physiological range, we chose this concentration to compare the effects of each peptide even if it has smaller effects. For pharmacological inhibition, the specific Gq inhibitor YM-254890 (Wako, Japan) was diluted to 100 nM in 0.01% dimethyl sulfoxide (DMSO) containing aCSF. Baseline TRH responses were recorded, followed by 40 minutes of incubation with YM-254890, a 20 minute aCSF wash, and a second TRH application. Notably, a washout step was not performed because YM-254890 binds irreversibly to Gq, making recovery of receptor activity impossible (30, 31).

### HCR *in situ* hybridization

*In situ* hybridization was performed using HCR™ technology (NEPA Gene, Chiba, Japan) following the manufacturer's instructions. The target genes were *trhra* (ENSORLG00000020692) and *pomca* (ENSORLG00000025908). The HCR probes and amplifier fluorophores used were as follows: *trhra* (S41-550, NEPA Gene) and *pomca* (S23-488, NEPA Gene). The sequences of the oligo probes used for each gene are listed in a table (Table S2 (32)). The brains were fixed by perfusion with 4% paraformaldehyde (PFA) for 2 hours at 4 °C, thereby removing blood cells showing strong autofluorescence. The samples were washed with PBS, equilibrated in 25% sucrose/PBS, and embedded in 5% LMT agarose containing 20% sucrose in PBS. Cryosections (25 μm) were prepared using a cryostat (Leica CM3050; objective −24 °C, chamber −28 °C) and mounted on CREST-coated slides (Matsunami, Kishiwada, Japan). Probe hybridization was performed according to the manufacturer's protocol. Excess probes were removed by washing with 5×saline-sodium citrate (SSC) buffer at 37 °C. Hairpins were gradually cooled, added to amplification buffer, and incubated at room temperature for 2 hours. After the excess hairpins were washed out, the sections were mounted for imaging and analysis.

### Reagents and resources

No antibodies or ELISA-based assays were used in this study.

### Data analysis

Changes in fluorescence during Ca^2+^ imaging were quantified using Fiji/ImageJ v1.54f. Statistical analyses were performed in Python 3.13.2 using NumPy, pandas, Matplotlib, SciPy, scikit-posthocs, and itertools. For each trace, fluorescence signals were normalized by the value at a defined baseline frame, such that ΔF/F was set to 1 at the start of the analysis window. Repeated-measures experiments (TRH, CRH, AVT) were analyzed using the Friedman test. Post hoc pairwise comparisons were performed using the Wilcoxon signed-rank test with Holm's correction for multiple comparisons. The data are presented as the means ± SEMs; statistical significance is indicated as follows: *P < .05, **P < .01, ***P < .001.

## Results

### Receptor expression in ACTH cells suggests candidate upstream regulators

**Figure 1.**
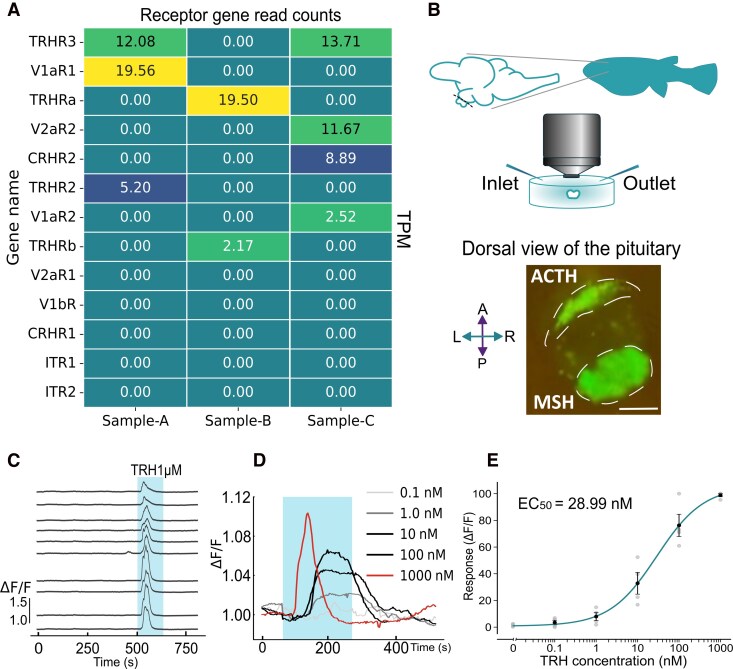
TRH induced activation of ACTH cells in the pituitary. (A) Transcriptome profiling of *pomca:GCaMP*–labeled ACTH cells, showing a transcript-per-million (TPM) list from preliminary RNA-seq analysis. This dataset represents a subset of the complete sequencing data presented in Fig. S1 (32). Sample names are consistent across this and Fig. S1 (32). (B) Schematic illustration of the Ca^2+^ imaging system. The fluorescence image shows the pituitary of a *pomca*:GCaMP transgenic medaka under epifluorescence fluorescence microscope, with ACTH cells located in the anterior region and MSH cells in the posterior region. Scale bar = 100 μm. (C) Representative single-cell traces of [Ca^2+^]ᵢ dynamics in ACTH cells during 1 μM TRH application. The shading indicates the perfusion period. (D) Concentration-dependent responses within the fish normalized to ΔF/F. Successive TRH applications at increasing concentrations elicited increasingly stronger [Ca^2+^]ᵢ responses. (E) Dose–response analysis across six concentrations of TRH, plotted as normalized ΔF/F_0_ responses of ACTH cells with a fitted dose–response curve. The fitted curve yielded an EC_50_ of 28.99 nM (95% CI: 23.86-34.13 nM). The data are presented as the mean ± SEM (n = 4 fish, 4 males).

Bulk RNA sequencing analysis (RNA-seq) of GCaMP-positive ACTH cells indicated the expression of receptor genes, providing clues for identifying upstream regulators of ACTH secretion. We isolated ACTH cells from dispersed pituitary *pomca*:GCaMP medaka cells under a fluorescence microscope and performed RNA-seq analysis. The specificity of GCaMP labeling has been reported previously (26), and before dispersion, we carefully removed the posterior part of the pituitary to minimize the contamination from MSH cells using a fluorescence dissection microscope. To exclude samples with amplification bias or contamination from other cell types, we performed quality control and selected datasets according to the criteria described in the Materials and Methods (Fig. S1A (32)). A list of gene IDs used in this analysis is provided in Table S1 (32). Among the samples that met these criteria, several candidate receptor genes for hypothalamic neuropeptides were detected (Fig. 1A). Based on these RNA-seq results of the receptor genes expressed in ACTH cells as well as previous studies (24, 25), we selected TRH, CRH, and AVT as candidate peptides for Ca^2+^ imaging analysis. It should be noted that these RNA-seq data were used for exploratory screening, as the samples may have contained transcripts from non-ACTH cell types; therefore, further validation is required for each candidate receptor.

### TRH induces [Ca^2+^]ᵢ elevation in pituitary ACTH cells

Ca^2+^ imaging was performed on pituitaries isolated from the brains of *pomca:*GCaMP transgenic medaka. These pituitaries allow a clear distinction between ACTH cells located in the rostral pars distalis and MSH cells in the pars intermedia (Fig. 1B). Analysis of [Ca^2+^]ᵢ dynamics of individual cells within the intact, isolated pituitary revealed that exposure to 1 μM TRH evoked synchronized increases in [Ca^2+^]ᵢ in multiple ACTH cells (Fig. 1C), and concentration-dependent responses were consistently observed in the ACTH cell-group region of interest (ROI) within fish (Fig. 1D). A dose–response analysis using 0-1000 nM TRH yielded an EC_50_ value of 28.99 nM (Fig. 1E, 95% confidence interval; CI: 23.86-34.13 nM), indicating that TRH effectively stimulates ACTH cells at concentrations comparable to those used in previous studies in mammals and teleosts (25, 33, 34). Collectively, these results demonstrate that TRH activates ACTH cells in the medaka pituitary in a dose-dependent manner within a physiological concentration range. Because ligands reach pituitary gradually in the bath perfusion preparation, higher TRH concentrations likely accelerate the apparent onset of responses by increasing diffusion-driven delivery to ACTH cells rather than by recruiting different receptor subtypes.

### TRH can activate ACTH cells via a Gq-dependent pathway, whereas CRH and AVT elicited no detectable [Ca^2+^]ᵢ responses

**Figure 2:**
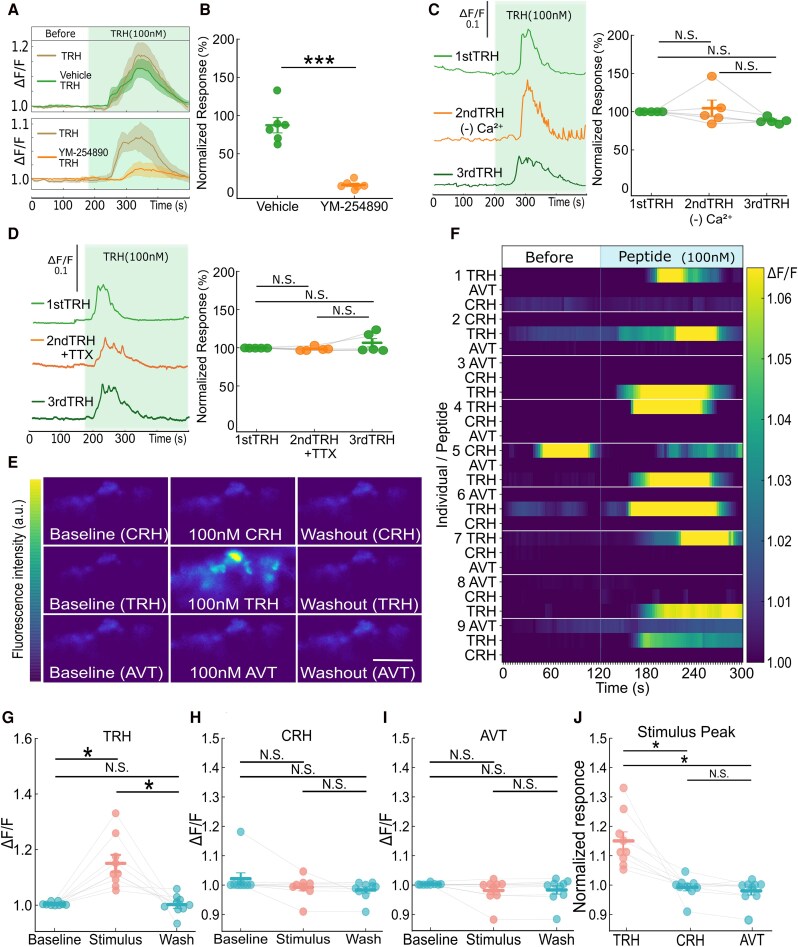
TRH, but not CRH or AVT, strongly stimulates ACTH cells. A-D; Inhibitor analysis of 100 nM TRH responses. (A) Summary of TRH-induced peak ΔF/F values before and after YM-254890 treatment (100 nM, 0.01% DMSO), shown as the mean ± SEM. Pituitaries were incubated with YM-254890 for 40 minutes and washed for 20 minutes in aCSF, and TRH responses were recorded again. The controls received DMSO alone. Statistical significance was assessed by unpaired Student's t test (***P < .001, n = 12 fish, male,4; female,8). (B) Normalized [Ca^2+^]ᵢ reponses to TRH in the presence of YM-254890, shown as the mean ± SEM. (C) [Ca^2+^]ᵢ responses to three consecutive TRH stimulations in ACTH cells, including the second stimulation under Ca^2+^-free conditions to confirm response reproducibility. The data are presented as the means ± SEMs; no significant differences were observed (N.S., n = 5 fish, male, 2; female,3). (D) To assess the effect of TTX within the same individual, we applied three consecutive TRH stimulations, with the second stimulation performed in the presence of TTX. The data are presented as the means ± SEMs; no significant differences were observed (N.S., n = 5 fish, male,3; female,2). (E) Representative image of ACTH cells baseline, during perfusion with 100 nM TRH, CRH, or AVT and washout, showing changes in fluorescence intensity. Scale bar = 100 μm. F-J: Quantification of peptide-induced responses in the same ROI. (F) Heatmaps of ACTH cells from all the fish, showing normalized ΔF/F responses during 100 nM TRH, CRH, or AVT perfusion. Peptide applications were performed in a randomized order, and the heatmaps are displayed in the same order. Reproducible [Ca^2+^]ᵢ increases were observed only during TRH perfusion. Relative changes in GCaMP fluorescence before, during, and after TRH(G) CRH (H) and AVT (I) perfusion. (J) Comparison of peak ΔF/F values induced by CRH, TRH, and AVT. Statistical significance was assessed using Friedman's test followed by the Wilcoxon signed-rank test and Holm's correction for multiple comparisons (*P < .05, n = 9 fish, male,6; female,3).

Because members of the TRH receptor family are known to couple to Gq proteins, we first examined the involvement of the Gq-mediated signaling pathway. Pituitaries were preincubated with the selective Gq inhibitor YM-254890 (100 nM, 0.01% DMSO; (35)) for 40 minutes, followed by a 20-minute washout before 100 nM TRH was applied. The control samples were treated with DMSO alone. Compared with control treatment, YM-254890 treatment markedly reduced TRH-induced [Ca^2+^]ᵢ responses (Fig. 2A and 2B), suggesting that TRH acts via a Gq-coupled G protein–coupled receptor (GPCR) pathway.

To determine whether TRH-induced [Ca^2+^]ᵢ responses depend on extracellular Ca^2+^ or synaptic input from residual axon terminals, 100 nM TRH was applied three times to the same cells in the presence of inhibitors in the isolated pituitary. During the second stimulation, the cells were perfused under Ca^2+^-free conditions (Fig. 2C) or in the presence of 1 μM tetrodotoxin (TTX; Fig. 2D). To compare repeated stimulations within the same cell, responses were normalized because each cell exhibited variation in overall fluorescence intensity. The peak amplitude of the first TRH response in each cell was defined as 100%, and subsequent responses were expressed relative to this value. Representative traces are also shown in the left panels (Fig. 2C and 2D). TRH-induced [Ca^2+^]ᵢ responses were not inhibited under either condition, indicating that ACTH cells respond to TRH independently of neural inputs from the nerve terminals of hypothalamic neurons. These results suggest that TRH can directly act on ACTH cells. Further work will be required to fully distinguish the relative contributions of intracellular Ca^2+^ store release and extracellular Ca^2+^ influx.

Next, we compared the effects of 100 nM TRH, CRH, and AVT on ACTH cell. Ca^2+^ imaging revealed robust and reproducible [Ca^2+^]ᵢ elevations only during TRH perfusion, whereas CRH and AVT elicited no detectable responses (Fig. 2E). Heatmaps summarizing normalized ΔF/F responses from multiple fish consistently showed strong activation only under TRH perfusion (Fig. 2F). The results from the quantitative analysis of the relative fluorescence changes confirmed that fluorescence significantly increased during TRH application, whereas CRH and AVT did not significantly change fluorescence levels (Fig. 2G-2I). Using the same data, we compared the effects of these peptides on the ACTH [Ca^2+^]ᵢ response within individual fish (Fig. 2J). These results suggest that in medaka TRH acts as a stronger stimulator of ACTH cells than CRH or AVT.

### TRH and CRH induce [Ca^2+^]ᵢ responses in MSH cells

**Figure 3:**
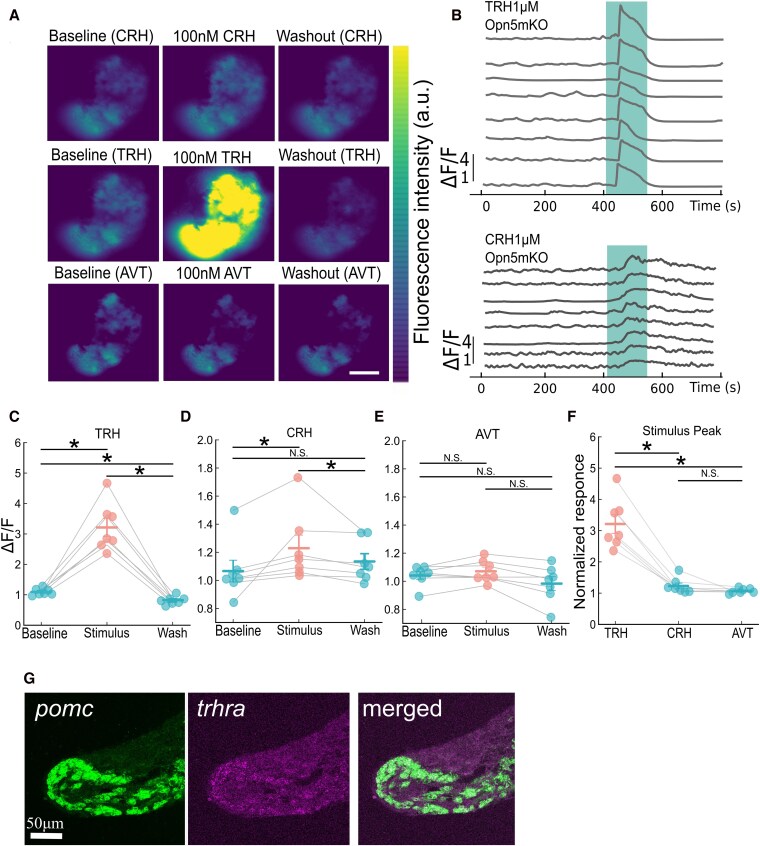
Effects of TRH and CRH on MSH cells. (A) Representative images of MSH cells at baseline, during perfusion with 100 nM TRH, CRH, or AVT and washout, showing changes in fluorescence intensity. Scale bar = 100 μm. (B) Individual [Ca^2+^]ᵢ traces of MSH cells during perfusion with 1 μM TRH or CRH, showing that MSH cells respond to both peptides, with TRH inducing rapid [Ca^2+^]ᵢ increases and CRH causing slower, more gradual increases than TRH does. C–F; Quantification of peptide-induced responses in MSH cells at a concentration of 100 nM. Peak ΔF/F values during TRH (C), CRH (D), or AVT (E) perfusion were compared within the same ROI, not across different cells (*P < .05, n = 7 fish, male,5; female,2). (F) Comparison of relative fluorescence changes induced by CRH, TRH, and AVT in the same ROI. The data are presented as the means ± SEMs. Statistical significance was assessed using Friedman's test followed by the Wilcoxon signed-rank test and Holm's correction for multiple comparisons (*P < .05, n = 7 fish, male,5; female,2). (G) Coronal pituitary sections (25 μm thick) were analyzed via *in situ* HCR, which revealed that MSH cells (*pomca*-positive) expressed *trhra* mRNA.

Following our analysis of ACTH cells, we next examined the effects of TRH, CRH, and AVT on MSH cells. To exclude Opn5m-mediated light-stimulated responses (26), we used isolated pituitaries from *pomca:GCaMP* transgenic *opn5m^−^/^−^* medaka for this imaging analysis. We perfused 100 nM TRH, CRH, or AVT and found that TRH strongly increased [Ca^2+^]ᵢ, whereas AVT did not alter the fluorescence intensity of GCaMP (Fig. 3A). Additionally, 1 μM TRH and CRH were also examined and the analysis of each cell was performed (Fig. 3B). Statistical analysis of the results of the perfusion experiments (100 nM) indicated that MSH cells responded to both TRH and CRH (Fig. 3C and 3D), whereas AVT perfusion did not significantly change the [Ca^2+^]ᵢ level (Fig. 3E). A comparison of peptides from the same dataset further revealed that TRH induced significant [Ca^2+^]ᵢ elevations in MSH cells compared with CRH or AVT (Fig. 3F). These results indicate that MSH cells respond to both TRH and CRH, with TRH inducing significantly greater [Ca^2+^]ᵢ elevations than CRH does. These findings also suggest that MSH secretion may be regulated by the integration of multiple stimuli. Since ovine CRH was used as a CRH receptor agonist in the experiments, we examined whether the effect of CRH is similar in its native peptide forms by using medaka CRHa and CRHb (Fig. S2A (32)). Here, as ovine CRH induced only small responses (Figs. 2E and 3H; 3B and 3D), we applied 400 nM CRH peptides, following the concentration in Fukuda et al (26). We found the [Ca^2+^]ᵢ response induced by medaka CRHa, CRHb, and ovine CRH showed no significant differences in either ACTH or MSH cells. Notably, subsequent application of 100 nM of TRH induced much more pronounced [Ca^2+^]ᵢ increase in both cell types compared to any of the CRH subtypes. Also, sequence alignment, and structural superposition of ovine CRH and medaka CRHa and CRHb peptides, indicated overall sequence and structural similarity, further supporting the use of ovine CRH as a suitable reference agonist. Sequence alignment showed high sequence similarity among ovine and medaka CRHs (Fig. S2B and S2C (32 )). Using the ChimeraX program for protein molecular visualization, we found very high structural similarity between ovine CRH and medaka CRHa and CRHb (Fig. S2C (32)). The average distance between the 3D structures of ovine and medaka CRH peptides (CRHa and CRHb) was less than 1 Å, indicating a high degree of structural similarity. The [Ca^2+^]ᵢ responses of ACTH and MSH cells were further characterized by analyzing the full width at half maximum, rise time, and decay half-time. These parameters were broadly similar between the two cell types, indicating comparable overall response kinetics (Fig. S3A (32)). We further compared the temporal profiles of the [Ca^2+^]ᵢ responses induced by CRH and TRH in MSH cells, with a focus on rise time, pre-peak response fraction, and decay half-time. The overall kinetics was largely comparable between the two stimuli; however, TRH responses tended to exhibit slightly shorter rise times, suggesting a tendency toward a more rapid increase in [Ca^2+^]ᵢ compared with CRH (Fig. S3B (32)). No formal statistical analysis was performed, and these observations are presented as descriptive comparisons.

### The expression of a TRH receptor was detected in MSH cells by *in situ* hybridization but was below the detection limit in ACTH cells

In addition, *in situ* hybridization chain reaction (*is*HCR) analysis of 25-μm-thick pituitary sections revealed that MSH cells (*pomca*-positive and located in the coronal section of the intermediate lobe) expressed *trhra* mRNA, indicating the presence of TRH receptors in MSH cells (Fig. 3G). In contrast, *trhr2* and *trhr3*, which were detected in ACTH cells by RNA-seq analysis, were not detectable by *is*HCR. This discrepancy can be attributable to differences in detection sensitivity between bulk transcriptomics and spatial *in situ* assays: low-abundance transcripts that are readily captured by RNA-seq often fall below the detection threshold of *is*HCR. Thus, while ACTH cells may express *trhr2* and/or *trhr3* at very low levels, such expression is likely insufficient for *in situ* visualization, and its functional relevance remains uncertain. On the other hand, unlike *in situ* visualization, RNA-seq may include inevitable contaminations of other cells. Therefore, we cannot conclude the presence or absence of the transcript without confirmation by *in situ* visualization. Together with our Ca^2+^ imaging data, these findings suggest that TRH responses in MSH cells are mediated by Trhra, whereas TRH-induced activation of ACTH cells may involve low-abundance receptor expression or alternative TRH-responsive pathways, both of which warrant further investigation.

## Discussion

### In medaka, TRH appears to be a more potent stimulator of ACTH and MSH secretion than the classically recognized hypothalamic regulators

In this study, we demonstrated that thyrotropin-releasing hormone (TRH) potently stimulates the release of two types of POMC-derived peptide hormones, ACTH and MSH. Interestingly, the synthetic TRH peptide showed more potent activation than ovine corticotropin-releasing hormone (CRH) and AVT did. Although the expression of multiple TRH receptor subtypes was not detected in ACTH cells by *is*HCR, given the presence of similar effects while inhibiting synaptic inputs with TTX, this finding suggests that TRH action on ACTH cells is likely direct. We also found that TRH stimulates MSH cells, which likely involves a cellular mechanism mediated by Trhra. Together, our data suggest that TRH regulates the release of both ACTH and MSH, two POMC-derived peptides. Although ACTH and MSH are generally considered to participate in separate physiological systems, namely the stress axis and the pigmentation pathway, our results suggest that hypothalamic TRH may act as a shared regulator that simultaneously controls both. Interestingly, body color is often associated with stress (36 ), which may be explained by the ligand-receptor crosstalk between ACTH and MSH systems (37). In addition to this, simultaneously regulated release of MSH and ACTH by the hypothalamic TRH may account for this phenomenon.

### TRH may act as a dominant secretagogue of ACTH cells in medaka

In this study, we visualized [Ca^2+^]ᵢ dynamics in medaka anterior pituitary ACTH cells using GCaMP6s and examined the effects of TRH, CRH, and AVT. We found that TRH consistently induced reproducible [Ca^2+^]ᵢ elevations in ACTH cells, whereas CRH and AVT elicited no significant changes (Fig. 2G-2J). Although CRH has been hypothesized to be a strong stimulator of ACTH secretion in vertebrates, these results suggest that TRH may be a dominant secretagogue in medaka. Furthermore, examinations using other fish will help clarify whether this phenomenon is a conserved mechanism or represents diversity within the teleost stress axis. A previous study has shown that 1 nM of ovine CRH stimulates ACTH release from the pars distalis of trout pituitaries and that the presence of AVT further synergistically enhances this effect (38). Similarly, ovine CRH perfusion induced ACTH release in gilthead sea bream (9, 10) and common carp (39), supporting the hypothesis that CRH acts as a primary hypothalamic regulator of ACTH secretion. However, in our medaka model, 100 nM CRH or AVT did not induce [Ca^2+^]ᵢ responses, which was consistent with the RNA-seq results showing no detectable expression of CRH receptor genes in ACTH cells (Fig. 1A). These differences may suggest the existence of species-specific receptor expression or the involvement of indirect regulatory pathways. Although transcriptomic datasets showed apparent expression of AVT receptor–related genes, these samples also exhibited elevated levels of other pituitary marker genes, suggesting sample-specific contamination or ambient RNA effects. Receptor reporter assays in medaka have demonstrated that V1a-type vasotocin receptors are capable of activating intracellular Ca^2+^ signaling via the PLC–IP_3_ pathway indicating that these receptors are functionally competent in this species (40). Therefore, if AVT receptors were robustly expressed in ACTH cells, AVT application would be expected to evoke [Ca^2+^]ᵢ responses. The absence of AVT-induced [Ca^2+^]ᵢ signals in our physiological experiments suggests that not only V1a-type receptors but Ca^2+^-mobilizing AVT receptors in general are minimally expressed or absent in ACTH cells. These findings highlight the importance of integrating physiological validation with transcriptomic detection when assessing cell-type–specific receptor expression.

Since we failed to detect TRH receptor expression in ACTH cells by *in situ* hybridization, it is difficult to determine the pathway by which TRH increases [Ca^2+^]ᵢ in ACTH cells. However, the following evidence suggests that TRH is highly likely to directly activate ACTH cells via some TRH receptors. First, TTX did not inhibit the TRH-induced [Ca^2+^]ᵢ increase in ACTH cells (Fig. 2D), indicating that this action does not require action-potential–dependent neuronal input. Notably, TTX can also block voltage-gated Na^+^ channels in endocrine cells, yet TRH responses persisted under this condition, demonstrating that voltage-gated Na^+^ channel activity is not required for the [Ca^2+^]ᵢ elevation. These observations indicate that TRH signaling operates independently of neuronal firing and Na^+^ channel–mediated excitability, even if indirect pathways such as gap junction coupling or paracrine signaling are involved.

Moreover, the TRH-induced [Ca^2+^]ᵢ elevation was blocked by the Gq inhibitor YM-254890, whereas Ca^2+^-free artificial cerebrospinal fluid (aCSF) did not alter the elevation, suggesting that this effect is mediated via a GPCR signaling pathway. Interestingly, three types of TRH receptors in Nile tilapia couple to both Gq and Gs pathways (41), whereas Gq inhibition alone abolished all [Ca^2+^]ᵢ responses in our study. This apparent discrepancy may suggest the involvement of mechanisms that we did not anticipate, including the possibility that TRH acts on ACTH cells indirectly or through an uncharacterized receptor. Further examination using other inhibitors of the Gq pathway is important to make a more solid conclusion regarding the cellular pathway. Although this study provides the direct observation of TRH-induced [Ca^2+^]ᵢ elevation in ACTH cells for the first time in teleosts, further studies are required to elucidate the molecular basis of TRH signaling, including the identification and localization of the TRH receptor subtype involved, as well as the potential contributions of other pituitary cell types.

### TRH may stimulate MSH release more potently than CRH does, and the TRH-induced response may be mediated by TRH receptor a (Trhra)

In contrast to ACTH cells, which responded specifically to TRH, MSH cells exhibited [Ca^2+^]ᵢ responses to both TRH and CRH (Fig. 3A-3E). Furthermore, *is*HCR analysis demonstrated the expression of *trhra* mRNA in *pomca*-positive MSH cells (Fig. 3F), suggesting that TRH likely acts directly on MSH cells through the Trhra receptor. In teleost species, there is direct anatomical evidence from axon tracing studies of TRH projections from the telencephalon (37) and preoptic area (42) to the pituitary, and immunohistochemical mapping has shown TRH immunoreactive (-TRH-ir) fibers in the pituitary (43, 44). These data provide an anatomical basis for the direct regulation of pituitary endocrine cells by TRH. Previous studies have also demonstrated that MSH release can be induced by both TRH (45, 46) and CRH (47, 48), while the expression of *crhr1* mRNA in the MSH cell-expressing area of medaka has been suggested (26). Our results are consistent with these findings in medaka and other species. However, in our analysis, the amplitude of the [Ca^2+^]ᵢ response was significantly greater with TRH stimulation than with ovine CRH at 100 nM (Fig. 3E), while medaka CRHa and CRHb elicited responses comparable to ovine CRH (Fig. S2A (32)), suggesting that TRH may serve a prominent role in MSH secretion in medaka.

### Multifactorial modulation of MSH secretion in fish: evolutionary and functional perspectives

MSH cells in medaka express the photoreceptor Opn5m and are known to exhibit [Ca^2+^]ᵢ elevation in response to light stimulation (26). Therefore, MSH secretion appears to be regulated by at least three types of inputs: TRH, CRH, and light. This suggested that MSH cells may function not only as passive targets of hypothalamic signals but also as integrative endocrine units that directly process multiple external stimuli. In mammals, except for the FSH and LH, which are produced in the same gonadotrophs, the secretion of specific pituitary hormones is fundamentally triggered by factors released from distinct hypothalamic neurons. Accordingly, these hypothalamic neurons function as central integrative hubs that synthesize diverse internal and external signals to determine pituitary endocrine output (49‐51). Evidence for such hypothalamic integration in teleosts is limited, but multiple hypothalamic peptides may act directly on specific pituitary cell types not limited to MSH cells, and similar organizational features have also been suggested in amphibians and reptiles (12, 16, 52, 53). These observations suggested that, unlike in mammals, some aspects of neuroendocrine integration may occur locally at the pituitary level. Together, multiple regulatory inputs to MSH cells in medaka may enable flexible modulation of pigmentation and other physiological processes in response to dynamic environmental and internal conditions. In this regard, future studies examining the synergistic effects of these three inputs, light, CRH, and TRH, might be of particular interest.

### Distinct hypothalamic pathways regulating ACTH secretion in vertebrates

TRH may directly activate ACTH cells in the pituitary of teleosts, suggesting the existence of a stress axis regulatory pathway that has not been previously recognized. CRH has been considered the primary factor driving ACTH secretion in vertebrates (54‐56); however, in amphibians, AVT has been reported to induce ACTH secretion more effectively than CRH does (13), indicating that the CRH-ACTH pathway is not uniformly conserved across all vertebrates. In non-mammalian vertebrates, CRH not only stimulates corticosteroid secretion but also exerts thyroid-stimulating effects, acting together with TRH to regulate the hypothalamic–pituitary–thyroid axis (15, 57, 58). In contrast, studies of TSH regulatory factors in fish (59) have indicated that TRH does not necessarily control TSH secretion. These findings suggest that multiple hypothalamic neural systems contribute to pituitary regulation and that their relative influence and dominant pathways may have changed over the course of evolution.

### Limitations and future directions

While our Ca^2+^ imaging assays revealed robust TRH-induced activation, direct evidence of ACTH secretion remains to be obtained. Future studies combining hormone release assays and receptor-specific knockouts (eg, *trhra*−/−, *trhrb*−/−, *trhr2*−/− and *trhr3*−/− lines) will help clarify the physiological contribution of TRH signaling to the stress response. We attempted to map TRH neuronal projections to ACTH and MSH cells using TRH-specific antibodies; however, neither of the two antibodies tested yielded specific labeling. In this study, as in previous reports (39, 60), we used ovine (sheep) CRH rather than native medaka CRH in the experiments shown in the main figures. However, as we compared the effects of ovine CRH and medaka CRH peptides and they indicated similar effects (Fig. S2 (32)), our results suggest that TRH exerts stronger functional effects than CRH in both ACTH and MSH cells in medaka. Furthermore, it remains unknown which populations of hypothalamic TRH neurons regulate each pituitary cell type. To address this, the generation of a *trh*:GFP transgenic medaka in combination with retrograde/anterograde tracer implantation will be needed to visualize TRH neuronal projections *in vivo*. Because ACTH cells are sparse and may alter gene expression in dissociated culture, current *in vitro* systems are not well suited to resolve direct vs indirect TRH actions. Addressing this question will require future studies using receptor-specific knockout models and improved culture methodologies. In addition, although dissociated primary pituitary cultures have been reported in teleosts, ACTH cells are sparse and show limited survival under current protocols, making reliable identification difficult. Endocrine cells may also alter hormone or receptor expression after isolation, complicating interpretation of direct vs indirect TRH effects.

## Conclusion

In summary, we demonstrate that TRH activates corticotropes and melanotropes in the medaka pituitary, more potently than well-known secretagogues such as CRH and AVT. These results reveal a previously unrecognized TRH-driven regulatory mechanism in the teleost stress axis and provide a foundation for understanding the evolutionary diversification of hypothalamic–pituitary communication in vertebrates.

## Data Availability

Some or all datasets generated during and/or analyzed during the current study are not publicly available but are available from the corresponding author on reasonable request.

## References

[1] Selye H . A syndrome produced by diverse nocuous agents. Nature. 1936;138(3479):32. 10.1176/jnp.10.2.230a 9722327

[2] Harris GW . Neural control of the pituitary gland.—I. Br Med J. 1951;2(4731):559‐564. 14869646 10.1136/bmj.2.4731.559 PMC2070089

[3] Saffran M, Schally AV. The release of corticotrophin by anterior pituitary tissue in vitro. Can J Biochem Physiol. 1955;33(1):408‐415. 14364332

[4] Guillemin R, Rosenberg B. Humoral hypothalamic control of anterior pituitary: a study with combined tissue cultures. Endocrinology. 1955;57(5):599‐607. 13270723 10.1210/endo-57-5-599

[5] Vale W, Spiess J, Rivier C, Rivier J. Characterization of a 41-residue ovine hypothalamic peptide that stimulates secretion of corticotropin and β-endorphin. Science. 1981;213(4514):1394‐1397. 6267699 10.1126/science.6267699

[6] Vale W, Rivier C, Brown MR, et al Chemical and biological characterization of corticotropin releasing factor. Recent Prog Horm Res. 1983;39:245‐270. 6314446 10.1016/b978-0-12-571139-5.50010-0

[7] Fryer JN, Lederis K. Control of corticotropin secretion in teleost fishes. Am Zool. 1986;26:1017‐1026.

[8] Balm P, Pepels P, Helfrich S, Hovens M, Bonga SW. Adrenocorticotropic hormone in relation to interrenal function during stress in tilapia (*Oreochromis mossambicus*). Gen Comp Endocrinol. 1994;96(3):347‐360. 7883141 10.1006/gcen.1994.1190

[9] Rotllant J, Balm PHM, Ruane NM, Pérez-Sánchez J, Wendelaar-Bonga SE, Tort L. Pituitary proopiomelanocortin-derived peptides and hypothalamus–pituitary–interrenal axis activity in gilthead sea bream (Sparus aurata) during prolonged crowding stress: differential regulation of adrenocorticotropin hormone and α-melanocyte-stimulating hormone release by corticotropin-releasing hormone and thyrotropin-releasing hormone. Gen Comp Endocrinol. 2000;119(2):152‐163. 10936035 10.1006/gcen.2000.7508

[10] Rotllant J, Balm P, Pérez-Sánchez J, Wendelaar-Bonga SE, Tort L. Pituitary and interrenal function in gilthead sea bream (Sparus aurata L., Teleostei) after handling and confinement stress. Gen Comp Endocrinol. 2001;121(3):333‐342. 11254375 10.1006/gcen.2001.7604

[11] Bernier NJ, Flik G, Klaren PHM. Chapter 6 Regulation and contribution of the corticotropic, melanotropic and thyrotropic axes to the stress response in fishes. In: Fish Physiology. 28: Academic Press; 2009:235‐311.

[12] Tonon MC, Cuet P, Lamacz M, et al Comparative effects of corticotropin-releasing factor, arginine vasopressin, and related neuropeptides on the secretion of ACTH and α-MSH by frog anterior pituitary cells and neurointermediate lobes in vitro. Gen Comp Endocrinol. 1986;61(3):438‐445. 3007273 10.1016/0016-6480(86)90231-5

[13] Okada R, Yamamoto K, Hasunuma I, Asahina J, Kikuyama S. Arginine vasotocin is the major adrenocorticotropic hormone-releasing factor in the bullfrog *Rana catesbeiana*. Gen Comp Endocrinol. 2016;237:121‐130. 27570059 10.1016/j.ygcen.2016.08.014

[14] Kaneko M, Fujisawa H, Okada R, Yamamoto K, Nakamura M, Kikuyama S. Thyroid hormones inhibit frog corticotropin-releasing factor-induced thyrotropin release from the bullfrog pituitary in vitro. Gen Comp Endocrinol. 2005;144(2):122‐127. 16040032 10.1016/j.ygcen.2005.05.003

[15] De Groef B, Van der Geyten S, Darras VM, Kühn ER. Role of corticotropin-releasing hormone as a thyrotropin-releasing factor in non-mammalian vertebrates. Gen Comp Endocrinol. 2006;146(1):62‐68. 16337947 10.1016/j.ygcen.2005.10.014

[16] Denver RJ, Licht P. Neuropeptides influencing in vitro pituitary hormone secretion in hatchling turtles. Journal of Experimental Zoology. 1989;251(3):306‐315.

[17] Carsia RV, Weber H, Perez FM Jr. Corticotropin-releasing factor stimulates the release of adrenocorticotropin from domestic fowl pituitary cells. Endocrinology. 1986;118(1):143‐148. 3000730 10.1210/endo-118-1-143

[18] Bu G, Fan J, Yang M, et al Identification of a novel functional corticotropin-releasing hormone (CRH2) in chickens and its roles in stimulating pituitary TSHβ expression and ACTH secretion. Front Endocrinol (Lausanne). 2019;10:595. 31555213 10.3389/fendo.2019.00595 PMC6727040

[19] Kuenzel WJ, Kang SW, Jurkevich A. Neuroendocrine regulation of stress in birds with an emphasis on vasotocin receptors (VTRs). Gen Comp Endocrinol. 2013;190:18‐23. 23500673 10.1016/j.ygcen.2013.02.029

[20] Karigo T, Aikawa M, Kondo C, Abe H, Kanda S, Oka Y. Whole brain-pituitary in vitro preparation of the transgenic medaka (*Oryzias latipes*) as a tool for analyzing the differential regulatory mechanisms of LH and FSH release. Endocrinology. 2014;155(2):536‐547. 24248459 10.1210/en.2013-1642

[21] Seale A, Fiess J, Hirano T, Cooke I, Grau E. Disparate release of prolactin and growth hormone from the tilapia pituitary in response to osmotic stimulation. Gen Comp Endocrinol. 2006;145(3):222‐231. 16242686 10.1016/j.ygcen.2005.09.006

[22] Fernandes GL, Gajbhiye DS, Columbus-Shenkar YY, Golan M. Novel tools for studying the fish growth hormone axis in vivo. Am J Physiol Endocrinol Metab. 2025;329(1):E131‐E142. 40490258 10.1152/ajpendo.00471.2024

[23] Tanaka S, Yu Y, Levavi-Sivan B, Zmora N, Zohar Y. GnRH—gonadotropes interactions revealed by pituitary single-cell transcriptomics in zebrafish. Endocrinology. 2024;165(12):bqae151. 39499852 10.1210/endocr/bqae151 PMC11565244

[24] Ager-Wick E, Maugars G, von Krogh K, Fontaine R, Weltzien F-A, Henkel C. An RNA-seq time series of the medaka pituitary gland during sexual maturation. Sci Data. 2023;10(1):62. 36720883 10.1038/s41597-023-01967-w PMC9889309

[25] Uehara SK, Nishiike Y, Maeda K, et al Identification of the FSH-RH as the other gonadotropin-releasing hormone. Nat Commun. 2024;15(1):5342. 38937445 10.1038/s41467-024-49564-8 PMC11211334

[26] Fukuda A, Sato K, Fujimori C, et al Direct photoreception by pituitary endocrine cells regulates hormone release and pigmentation. Science. 2025;387(6729):43‐48. 39745961 10.1126/science.adj9687

[27] Dobin A, Davis CA, Schlesinger F, et al STAR: ultrafast universal RNA-seq aligner. Bioinformatics. 2013;29(1):15‐21. 23104886 10.1093/bioinformatics/bts635 PMC3530905

[28] Li B, Dewey CN. RSEM: accurate transcript quantification from RNA-seq data with or without a reference genome. BMC Bioinformatics. 2011;12(1):323. 21816040 10.1186/1471-2105-12-323 PMC3163565

[29] Bernier NJ, Flik G, Klaren PH. Regulation and contribution of the corticotropic, melanotropic and thyrotropic axes to the stress response in fishes. Fish Physiol. 2009;28:235‐311.

[30] Nishimura A, Kitano K, Takasaki J, et al Structural basis for the specific inhibition of heterotrimeric Gq protein by a small molecule. Proc Natl Acad Sci U S A. 2010;107(31):13666‐13671. 20639466 10.1073/pnas.1003553107 PMC2922266

[31] Schrage R, Schmitz A-L, Gaffal E, et al The experimental power of FR900359 to study Gq-regulated biological processes. Nat Commun. 2015;6(1):10156. 26658454 10.1038/ncomms10156 PMC4682109

[32] Yamakawa M, Gajbhiye DS, Golan M, Kanda S. Supplementary data for: TRH can stimulate the release of two POMC-derived pituitary hormones, ACTH and MSH, in medaka. Zenodo. 2026. 10.5281/zenodo.18849968 PMC13112426 41913960

[33] Balik A, Jindrichova M, Bhattacharyya S, Zemkova H. GnRH-I and GnRH-II-induced calcium signaling and hormone secretion in neonatal rat gonadotrophs. Physiol Res. 2009;58(5):709‐716. 19093727 10.33549/physiolres.931632

[34] Rousseau BL, Marchelidon D. Evidence that corticotropin-releasing hormone acts as a growth hormone-releasing factor in a primitive teleost, the European eel (*Anguilla Anguilla*). J Neuroendocrinol. 1999;11(5):385‐392. 10320566 10.1046/j.1365-2826.1999.00334.x

[35] Mamedova E, Dmytriyeva O, Rekling JC. Thyrotropin-releasing hormone induces Ca2 + increase in a subset of vagal nodose ganglion neurons. Neuropeptides. 2022;94:102261. 35704969 10.1016/j.npep.2022.102261

[36] Kittilsen S, Schjolden J, Beitnes-Johansen I, et al Melanin-based skin spots reflect stress responsiveness in salmonid fish. Horm Behav. 2009;56(3):292‐298. 19539629 10.1016/j.yhbeh.2009.06.006

[37] Khan UW, Øverli Ø, Hinkle PM, et al A novel role for pigment genes in the stress response in rainbow trout (*Oncorhynchus mykiss*). Sci Rep. 2016;6(1):28969. 27373344 10.1038/srep28969 PMC4931468

[38] Baker BI, Bird DJ, Buckingham JC. In the trout, CRH and AVT synergize to stimulate ACTH release. Regul Pept. 1996;67(3):207‐210. 8988522 10.1016/s0167-0115(96)00130-9

[39] Metz JR, Huising MO, Meek J, Taverne-Thiele AJ, Wendelaar Bonga SE, Flik G. Localization, expression and control of adrenocorticotropic hormone in the nucleus preopticus and pituitary gland of common carp (*Cyprinus carpio* L.). J Endocrinol. 2004;182(1):23‐31. 15225128 10.1677/joe.0.1820023

[40] Yamaguchi Y, Kaiya H, Konno N, et al The fifth neurohypophysial hormone receptor is structurally related to the V2-type receptor but functionally similar to V1-type receptors. Gen Comp Endocrinol. 2012;178(3):519‐528. 22809669 10.1016/j.ygcen.2012.07.008

[41] Bu G, Yong T, Tang Y, et al Characterization of thyrotropin-releasing hormone (TRH) and its receptors (TRHRs) in Nile Tilapia: molecular identification, ligand-receptor interaction and expression profile. Peptides. 2025;19:171426. 10.1016/j.peptides.2025.171426 40614932

[42] Singh O, Pradhan DR, Nagalakashmi B, et al Thyrotropin-releasing hormone (TRH) in the brain and pituitary of the teleost, *Clarias batrachus* and its role in regulation of hypophysiotropic dopamine neurons. J Comp Neurol. 2019;527(6):1070‐1101. 30370602 10.1002/cne.24570

[43] Díaz ML, Becerra M, Manso MJ, Anadón R. Distribution of thyrotropin-releasing hormone (TRH) immunoreactivity in the brain of the zebrafish (*Danio rerio*). J Comp Neurol. 2002;450(1):45‐60. 12124766 10.1002/cne.10300

[44] Batten T, Moons L, Cambre M, Vandesande F, Seki T, Suzuki M. Thyrotropin-releasing hormone-immunoreactive system in the brain and pituitary gland of the sea bass (*Dicentrarchus labrax*, Teleostei). Gen Comp Endocrinol. 1990;79(3):385‐392. 2125566 10.1016/0016-6480(90)90068-w

[45] Tran TN, Fryer JN, Bennett HPJ, Tonon MC, Vaudry H. TRH stimulates the release of POMC-derived peptides from goldfish melanotropes. Peptides. 1989;10(4):835‐841. 2555798 10.1016/0196-9781(89)90122-8

[46] Lamers AE, Flik G, Bonga S. A specific role for TRH in release of diacetyl alpha-MSH in tilapia stressed by acid water. Am J Physiol. 1994;267(5):R1302‐R1308. 7977858 10.1152/ajpregu.1994.267.5.R1302

[47] Van den Burg E, Metz J, Spanings F, Bonga SW, Flik G. Plasma α-MSH and acetylated β-endorphin levels following stress vary according to CRH sensitivity of the pituitary melanotropes in common carp, *Cyprinus carpio*. Gen Comp Endocrinol. 2005;140(3):210‐221. 15639149 10.1016/j.ygcen.2004.11.010

[48] Huising M, Van Schooten C, Taverne-Thiele A, Hermsen T, Verburg-van Kemenade B, Flik G. Structural characterisation of a cyprinid (*Cyprinus carpio* L.) CRH, CRH-BP and CRH-R1, and the role of these proteins in the acute stress response. J Mol Endocrinol. 2004;32(3):627‐648. 15171705 10.1677/jme.0.0320627

[49] Saper CB, Lowell BB. The hypothalamus. Curr Biol. 2014;24(23):R1111‐R1116. 25465326 10.1016/j.cub.2014.10.023

[50] Chen X, Wang Y, Fu S, et al The integrated function of the lateral hypothalamus in energy homeostasis. Cells. 2025;14(14):1042. 40710295 10.3390/cells14141042 PMC12293592

[51] Coll AP, Yeo GS. The hypothalamus and metabolism: integrating signals to control energy and glucose homeostasis. Curr Opin Pharmacol. 2013;13(6):970‐976. 24075719 10.1016/j.coph.2013.09.010

[52] Fryer J, Lederis K, Rivier J. ACTH-releasing activity of urotensin I and ovine CRF: interactions with arginine vasotocin, isotocin and arginine vasopressin. Regul Pept. 1985;11(1):11‐15. 2989979 10.1016/0167-0115(85)90026-6

[53] Fryer JN . Neuropeptides regulating the activity of goldfish corticotropes and melanotropes. Fish Physiol Biochem. 1989;7(1-6):21‐27. 24221751 10.1007/BF00004686

[54] Flik G, Klaren PH, Van den Burg EH, Metz JR, Huising MO. CRF and stress in fish. Gen Comp Endocrinol. 2006;146(1):36‐44. 16403502 10.1016/j.ygcen.2005.11.005

[55] Denver RJ . Structural and functional evolution of vertebrate neuroendocrine stress systems. Ann N Y Acad Sci. 2009;1163(1):1‐16. 19456324 10.1111/j.1749-6632.2009.04433.x

[56] Lovejoy DA, Chang BS, Lovejoy NR, del Castillo J. Molecular evolution of GPCRs: CRH/CRH receptors. J Mol Endocrinol. 2014;52(3):T43‐T60. 24711645 10.1530/JME-13-0238

[57] De Groef B, Geris K, Vandenborne K, Darras V, Kühn E. CRH control of Thyroid Function in the Chicken. Functional Avian Endocrinology Narosa Publishing House; 2005:415‐426.

[58] De Groef B, Vandenborne K, Van As P, et al Hypothalamic control of the thyroidal axis in the chicken: over the boundaries of the classical hormonal axes. Domest Anim Endocrinol. 2005;29(1):104‐110. 15927770 10.1016/j.domaniend.2005.02.008

[59] Larsen DA, Swanson P, Dickey JT, Rivier J, Dickhoff WW. In vitro thyrotropin-releasing activity of corticotropin-releasing hormone-family peptides in *Coho Salmon*, *Oncorhynchus kisutch*. Gen Comp Endocrinol. 1998;109(2):276‐285. 9473372 10.1006/gcen.1997.7031

[60] Fryer J, Lederis K, Rivier J. Urotensin I, a CRF-like neuropeptide, stimulates ACTH. Release from the teleost pituitary. Endocrinology. 1983;113(6):2308‐2310. 6315348 10.1210/endo-113-6-2308

